# (−)-Agelasidine A Induces Endoplasmic Reticulum Stress-Dependent Apoptosis in Human Hepatocellular Carcinoma

**DOI:** 10.3390/md20020109

**Published:** 2022-01-29

**Authors:** I-Ta Lu, Shih-Chao Lin, Yi-Chia Chu, Ya Wen, You-Cheng Lin, Wen-Chien Cheng, Jyh-Horng Sheu, Chi-Chien Lin

**Affiliations:** 1 Program in Translational Medicine, National Chung Hsing University, Taichung 402, Taiwan; luita@vghtc.gov.tw (I.-T.L.); d14321@mail.cmuh.org.tw (W.-C.C.); 2 Division of Gastroenterology, Taichung Veterans General Hospital, Taichung 407, Taiwan; 3 Bachelor Degree Program in Marine Biotechnology, College of Life Sciences, National Taiwan Ocean University, Keelung 202, Taiwan; sclin@mail.ntou.edu.tw; 4 Institute of Biomedical Science, The iEGG and Animal Biotechnology Center, National Chung-Hsing University, Taichung 402, Taiwan; girl770409@smail.nchu.edu.tw; 5 Department of Physiology and Pharmacology, Karolinska Institutet, SE-171 77 Stockholm, Sweden; ya.wen@ki.se; 6 Doctoral Degree Program in Marine Biotechnology, National Sun Yat-sen University, Kaohsiung 804, Taiwan; d045620002@nsysu.edu.tw; 7 Division of Pulmonary and Critical Care Medicine, China Medical University, Hospital, Taichung 407, Taiwan; 8 Department of Marine Biotechnology and Resources, National Sun Yat-sen University, Kaohsiung 804, Taiwan; 9 Graduate Institute of Natural Products, Kaohsiung Medical University, Kaohsiung 807, Taiwan; 10 Department of Medical Research, China Medical University Hospital, Taichung 404, Taiwan; 11 Department of Medical Research, Taichung Veterans General Hospital, Taichung 407, Taiwan; 12 Department of Pharmacology, College of Medicine, Kaohsiung Medical University, Kaohsiung 807, Taiwan; 13 Department of Biotechnology, Asia University, Taichung 413, Taiwan

**Keywords:** hepatocellular carcinoma (HCC), (−)-agelasidine A, apoptosis, caspase, death receptor, mitochondrial membrane potential, endoplasmic reticulum stress

## Abstract

Liver cancers, such as hepatocellular carcinoma (HCC), are a highly prevalent cause of cancer-related deaths. Current treatments to combat liver cancer are limited. (−)-Agelasidine A, a compound isolated from the methanol extract of *Agelas* *nakamurai*, a sesquiterpene guanidine derived from sea sponge, has antibacterial activity. We demonstrated its anticancer capabilities by researching the associated mechanism of (−)-agelasidine A in human liver cancer cells. We found that (−)-agelasidine A significantly reduced viability in Hep3B and HepG2 cells, and we determined that apoptosis was involved in the (−)-agelasidine A-induced Hep3B cell deaths. (−)-Agelasidine A activated caspases 9, 8, and 3, as well as PARP. This effect was reversed by caspase inhibitors, suggesting caspase-mediated apoptosis in the (−)-agelasidine A-treated Hep3B cells. Moreover, the reduced mitochondrial membrane potential (MMP) and the release of cytochrome c indicated that the (−)-agelasidine A-mediated mitochondrial apoptosis was mechanistic. (−)-Agelasidine A also increased apoptosis-associated proteins (DR4, DR5, FAS), which are related to extrinsic pathways. These events were accompanied by an increase in Bim and Bax, proteins that promote apoptosis, and a decrease in the antiapoptotic protein, Bcl-2. Furthermore, our results presented that (−)-agelasidine A treatment bridged the intrinsic and extrinsic apoptotic pathways. Western blot analysis of Hep3B cells treated with (−)-agelasidine A showed that endoplasmic reticulum (ER) stress-related proteins (GRP78, phosphorylated PERK, phosphorylated eIF2α, ATF4, truncated ATF6, and CHOP) were upregulated. Moreover, 4-PBA, an ER stress inhibitor, could also abrogate (−)-agelasidine A-induced cell viability reduction, annexin V+ apoptosis, death receptor (DR4, DR5, FAS) expression, mitochondrial dysfunction, and cytochrome c release. In conclusion, by activating ER stress, (−)-agelasidine A induced the extrinsic and intrinsic apoptotic pathways of human HCC.

## 1. Introduction

Primary liver cancer, mainly hepatocellular carcinoma (HCC), is the fourth leading cause of worldwide cancer deaths, accounting for an estimated 780,000 deaths annually [1,2]. Hepatocellular carcinoma (HCC) is the most prevalent subtype responsible for liver cancer death. Despite advances in diagnosis and treatment, most HCC patients are diagnosed at advanced stages when predicted survival is less than 1 year [3,4]. Thus, novel therapeutic agents against HCC are urgently required.

Bioactive compounds derived from natural products are promising candidates for cancer drug development [5,6]. In the past four decades, natural anticancer products accounted for approximately 15.4% of new molecular drugs [7]. Marine organism-produced natural compounds have multifunctional biological activities and provide a new pool of potential cancer treatment options [8,9]. Cytarabine, the first marine derivative discovered 50 years ago, was originally isolated from a sponge and was approved by the FDA for the treatment of leukemia in 1969 [10].

In recent years, bioactive marine alkaloids have been found to be effective in several therapeutic fields such as antibacterial, antioxidant, and anticancer [11]. Among alkaloids, guanidine is a strong base, which has become a key motif of many clinical drugs such as the famous antidiabetic drug, metformin (dimethyl-biguanide), and the drug for treating peptic ulcers, cimetidine. In terms of anticancer effects, previous in vitro studies have shown that guanidine marine alkaloids have potential anticancer properties and cytotoxic activities [12]. Crambescidin-816 is a pentacyclic guanidine alkaloid isolated from *Crambe crambe* (oyster sponge), which could reduce the cell viability of liver cancer cells in experiments [13].

Marine sponges of the *Agelas* genus were found to be prolific producers of bromopyrrole derivatives and terpenoid alkaloids. The first isolated derivative and a major sesquiterpene alkaloid possessing sulfone and guanidine functional groups, agelasidine A, was discovered and reported by Nakamura et al. in 1983 from *Agelas* sp. This organism was identified as *Agelas nakamurai* in a related article published in 1985, with the value of specific optical rotation being +19.1 [14,15]. Subsequently, the same metabolite referred to as agelasidine A or, more specifically, (+)-agelasidine A was again isolated from a sponge of the same genus of an unknown species in 1984 by Capon et al. [16]. (+)-Agelasidine A has been found to display antispasmodic and antibacterial activities [16]. In 2006, the optical isomer (−)-agelasidine A, with the value of specific optical rotation being −12.2, was discovered for the first time from the sponge *A. clathrodes* by Medeiros et al. [17]. However, so far, there are still very few studies focusing on the biological activity of (−)-agelasidine A, except for previous studies that proved its antibacterial effect [17]. In addition, (−)-agelasidine A challenge against cancer has not been explored in the currently available literature. To expand knowledge of this compound, we determined the cytotoxic effects of (−)-agelasidine A isolated from *Agelas* *nakamurai* in human Hep3B and HepG2 liver cancer cell lines. In this paper, we also explored how (−)-agelasidine A induced cancer cell death.

## 2. Results

### 2.1. (−)-Agelasidine A Inhibited Cell Growth and Promoted Apoptosis in Human Liver Cancer Cells

**Figure 1:**
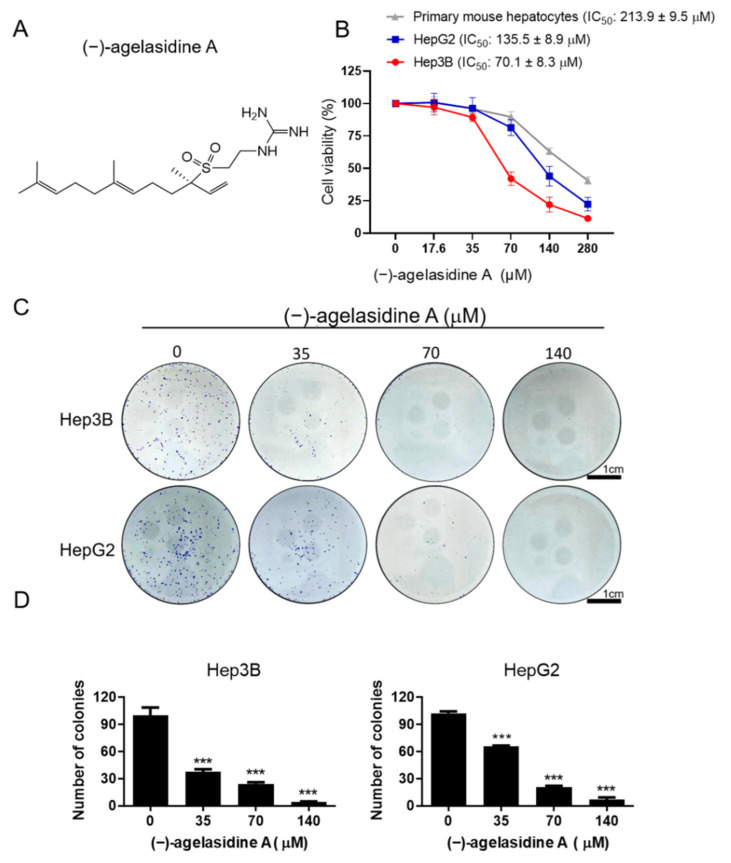
(**A**) The effect of (−)-agelasidine A on cell viability and colony formation in HePG2, Hep3B, and primary mouse hepatocyte cells. Cells were treated with 0.1% DMSO (0 μM), or (−)-agelasidine A at 17.6, 35, 70, 140, or 280 μM for 24 h. (**B**) Cell viability was examined by MTT assay. (**C**,**D**) Colony formation assay of HepG2 and HeP3B cells following treatment with (−)-agelasidine A for 1 week. Data are presented as the mean ± SD in four biological replicates for each treatment. Significant differences compared to the 0.1% DMSO control group are indicated by *** *p* < 0.001.

(−)-agelasidine A (139.7 mg) has been isolated from the frozen *Agelas nakamurai* (294.0 g). To test the ability of (−)-agelasidine A to inhibit cancer cell growth, we performed cell viability assays on two liver cancer cell lines, HepG2 and Hep3B, as well as primary murine hepatocytes, with and without the treatment of (−)-agelasidine A (Figure 1A). Specifically, cells were directly added with various concentrations of (−)-agelasidine A followed by cell survival determination using the MTT assay. With the data from MTT assays, we determined the IC_50_ values for HepG2, Hep3B, and primary mouse hepatocytes treated with (−)-agelasidine A as 129.8 ± 7.3, 69.9 ± 6.4, and >280 μM, respectively (Figure 1B). The results showed that the compound was capable of decreasing cancer cell viability in a dose-dependent manner and was more detrimental toward cancerous cells than primary normal hepatocytes. As further confirmation, colony formation assays were performed, treating for 1 week with (−)-agelasidine A; once again, dose-dependent inhibition of Hep3B and HepG2 was shown (Figure 1C,D). These results confirmed the growth-inhibitory effect of (−)-agelasidine A. Our data indicated that Hep3B cells were more sensitive to (−)-agelasidine A than HepG2 cells. From these results, we determined that Hep3B cells represented a sufficient model for (−)-agelasidine A treatment studies.

### 2.2. (−)-Agelasidine A Initiated Apoptosis in-Hep3B Cells

**Figure 2:**
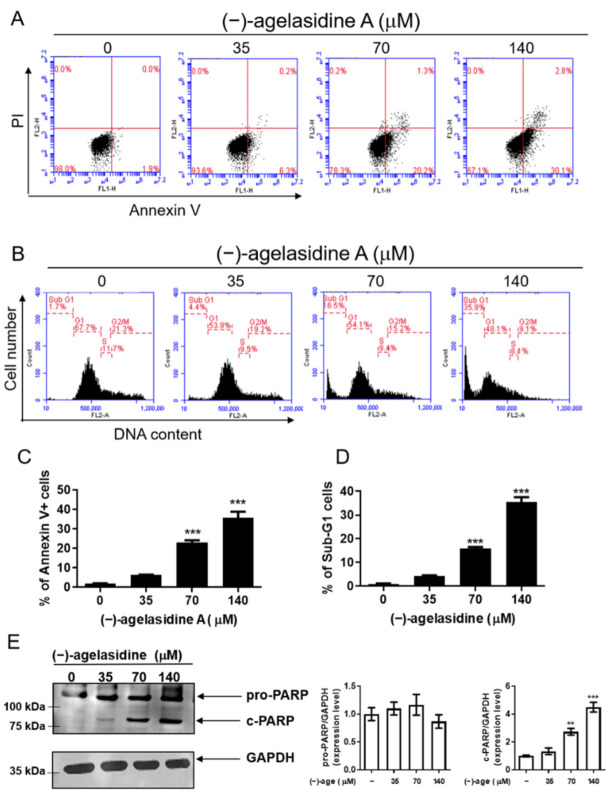
The effect of (−)-agelasidine A on apoptosis in Hep3B cells. Cells were treated with 0.1% DMSO (0 μM) or (−)-agelasidine A at 35, 70, or 140 μM for 24 h. (**A**,**C**) Phosphatidylserine externalization and DNA integrity were determined by FITC/annexin V and PI, respectively. (**B**,**D**) Cell-cycle distributions were determined by propidium iodide (PI) staining and flow cytometry analysis. The means ± SEM of the experimental triplicates are presented in the bar graph at the bottom. (**E**) Expression levels of cleaved poly (ADP-ribose) polymerase (PARP) were investigated by Western blotting using GAPDH as a loading control. Three biological replicates were conducted in each experiment, and representative graphs and photos from one of three independent experiments with similar results are presented. Significant differences compared to the 0.1% DMSO-treated control group are indicated by ** *p* < 0.01, *** *p* < 0.001.

To further clarify the cytotoxic effect of (−)-agelasidine A, apoptosis and cell-cycle distribution studies were conducted using annexin V/FITC and propidium iodide staining, along with flow cytometry. Hep3B cells were treated with varying doses of (−)-agelasidine A for 24 h. As shown in Figure 2A,C, as the dose increased, we witnessed an increase in the number of annexin V-positive cells. There was also a detectable increase in sub-G1 populations as the dose increased, further supporting increased cell death caused by (−)-agelasidine A (Figure 2B,D). Activation of Poly (ADP-ribose) polymerase (PARP) is well known as a marker of apoptotic cells. Therefore, we detected the expression of cleaved PARP with Western blotting and found cleavage of PARP to increase as (−)-agelasidine A dose increased in Hep3B cells (Figure 2E). These results suggest that (−)-agelasidine A induces apoptosis in Hep3B cells. The data are presented as the mean ± SD from at least three replicates.

### 2.3. (−)-Agelasidine A Induced Hep3B Cell Apoptosis through a Caspase-Dependent Pathway

**Figure 3:**
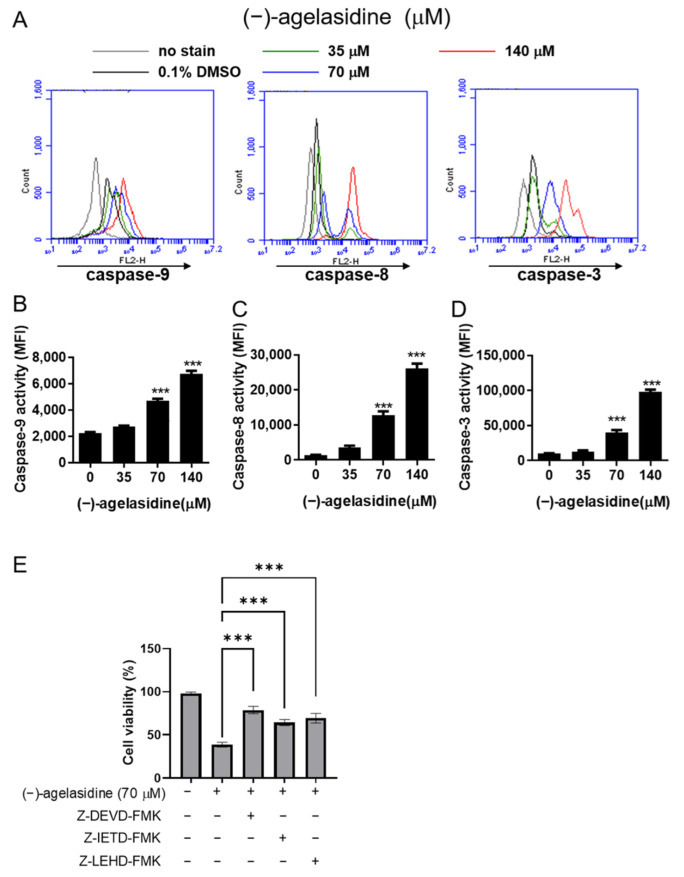
The effects of (−)-agelasidine A on caspase-3, -8, and -9 activities in HEP3B cells. HEP3B cells were treated with different concentrations of (−)-agelasidine A for 24 h, and the activities of (**A**) caspase-9, (**B**) caspase-8, and (**C**) caspase-3 were determined via flow cytometry. (**D**,**E**) The bar data represent the means ± SD of samples from three wells from one of three independent experiments with similar results. Significant differences compared to the DMSO-treated control group are indicated by asterisks (*** *p* < 0.001).

Activities of caspase-9, caspase-8, and caspase-3 were tested to explore the effects (−)-agelasidine A on the caspase-mediated pathway. As shown in Figure 3, caspase-3 (Figure 3A,B), caspase-8 (Figure 3A,C), and caspase-9 (Figure 3A,D) activities escalated in response to increasing doses of (−)-agelasidine A. To confirm the role of caspases, we attempted to suppress each caspase activity with individual inhibitors, Z-DEVD-FMK, Z-IETD-FMK, and Z-LEHD-FMK, which have distinct inhibitory effects on caspases-3, -8, and -9, respectively, followed by post-treatment with (−)-agelasidine A, to determine the imperative role of the caspase pathway. As shown in Figure 3E, pretreatment of Hep3B cells with each individual caspase inhibitor significantly increased the viability of the cells subsequently treated with (−)-agelasidine A. These data strongly suggest that caspase-mediated apoptosis contributed to (−)-agelasidine A-induced growth inhibition.

### 2.4. (−)-Agelasidine A Induced Mitochondrial Dysfunction in Hep3B Cells

**Figure 4:**
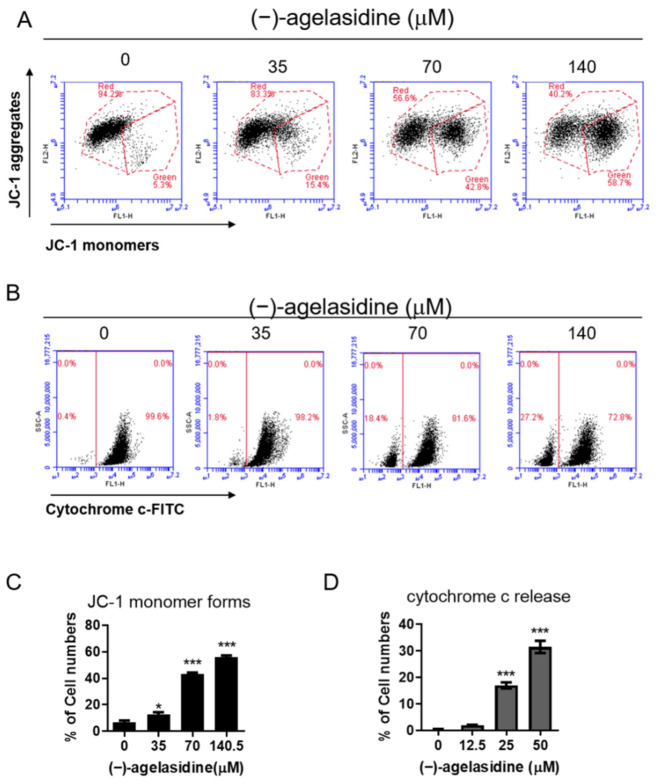
The effects of (−)-agelasidine A on mitochondrial dysfunction in Hep3B Cells. Cells were treated with different concentrations of (−)-agelasidine A for 24 h. (**A**) Mitochondrial membrane potential (Δψm) and (**B**) cytochrome C release were determined by JC-1 fluorescent dye staining or anti-cytochrome c –FITC antibody and flow cytometry analysis. The means ± SD of the experimental triplicates are presented in the bar graphs of the (**C**) JC-1 aggregates and (**D**) cytochrome c release. All data presented are representative of three independent experiments with similar results. Significant differences compared to the 0.1% DMSO-treated control group are indicated by * *p* < 0.05, *** *p* < 0.001.

**Figure 5:**
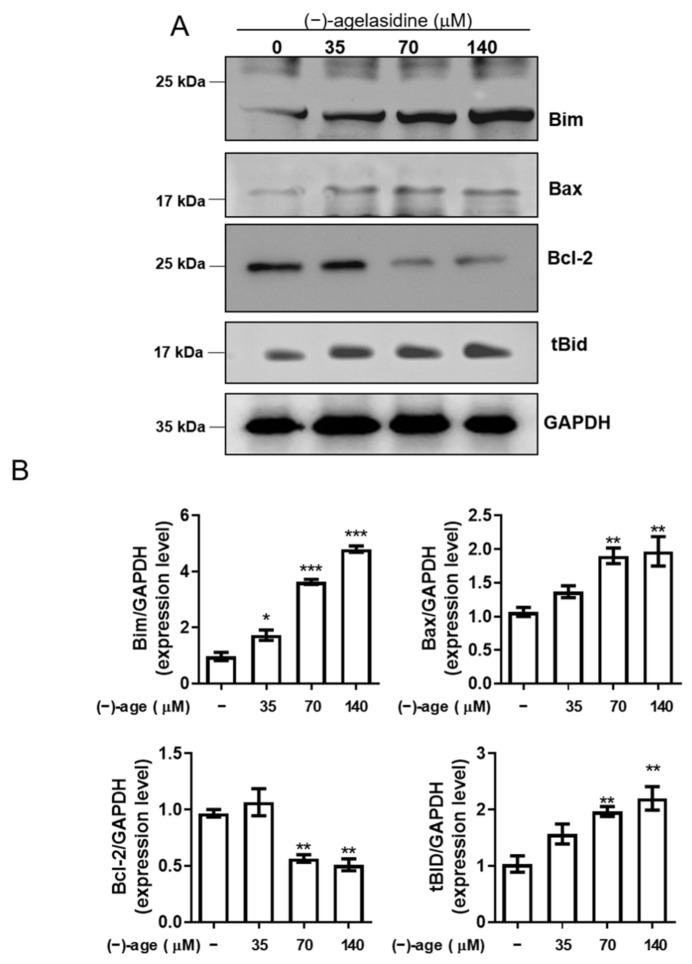
The effects of (−)-agelasidine A on the expression of Bcl-2 family in Hep3B cells. Cells were treated with different concentrations of (−)-agelasidine A for 24 h. (**A**) The Bim, Bax, Bcl-2, and truncated Bid (tBID) levels were examined by Western blot analysis. GAPDH was used as a loading control. (**B**) Quantitation of Western blot signal intensities. Data from three replicates were presented, and experiments were repeated three times with similar results. Significant differences compared to the DMSO-treated control group are indicated by * *p* < 0.05, ** *p* < 0.01, *** *p* < 0.001.

We tested whether (−)-agelasidine A-induced apoptosis was associated with mitochondrial dysfunction. As can be seen from the results of JC-1 staining, the mitochondrial membrane potential (MMP)-dependent formation of JC-1 aggregation in mitochondria was maintained at a relatively high rate in Hep3B cells not treated with (−)-agelasidine A (Figure 4A,C). However, JC-1 monomers increased after treatment with (−)-agelasidine A in a concentration-dependent manner, indicating a significant depletion of MMP after (−)-agelasidine A treatment. As indicated in Figure 4B,D, we also found that mitochondrial release of cytochrome c was promoted in (−)-agelasidine A-treated Hep3B cells. (−)-Agelasidine A also increased the expression of proapoptotic Bim and Bax, but decreased the expression of antiapoptotic Bcl-2 (Figure 5A,B). Additionally, the expression of truncated BH3 interacting-domain death (tBid) agonist-was increased (Figure 5A,B). These data demonstrate that (−)-agelasidine A-induced apoptosis was involved in the mitochondrial execution pathway in Hep3B cells.

### 2.5. (−)-Agelasidine A Induced Death Receptor Expression in Hep3B Cells

**Figure 6:**
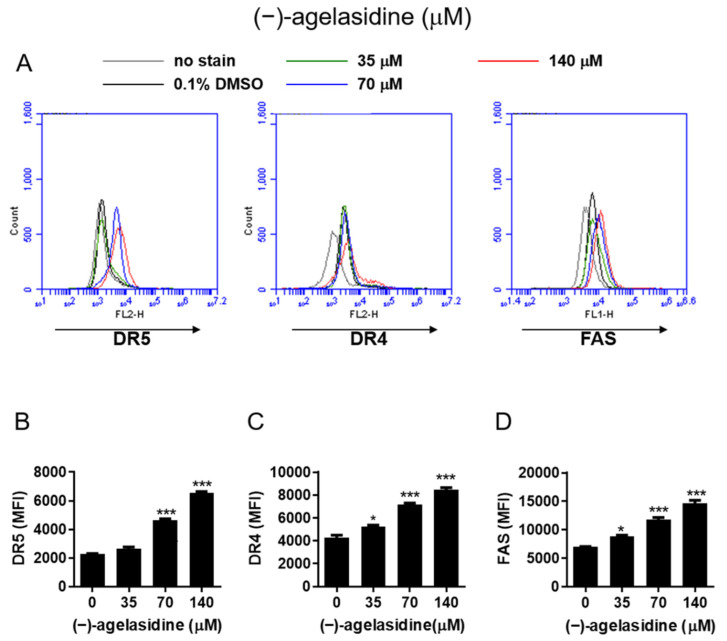
The effects of (−)-agelasidine A on death receptor DR5, DR4, and FAS expression in Hep3B cells. HEP3B cells were treated with different concentrations of (−)-agelasidine A for 24 h, and the activities of (**A**) DR5, DR4, and FAS were determined via flow cytometry. (**B**–**D**) The bar data represent the means ± SD of samples from three wells. Data are presented as the mean ± SD in three replicates for each treatment. All data presented are representative of three independent experiments with similar results. Significant differences compared to the DMSO-treated control group are indicated by * *p* < 0.05, *** *p* < 0.001.

Since (−)-agelasidine A induced caspase-8 activation (Figure 3A) and increased the amount of tBid (Figure 5A,B), we further investigated the effect of (−)-agelasidine A on the surface expression of DR4, DR5, and FAS proteins. In the Hep3B cell line, (−)-agelasidine A increased the expression of DR4, DR5, and FAS (Figure 6A–D) at the protein level.

### 2.6. (−)-Agelasidine A Activated Endoplasmic Reticulum Stress in Hep3B

**Figure 7:**
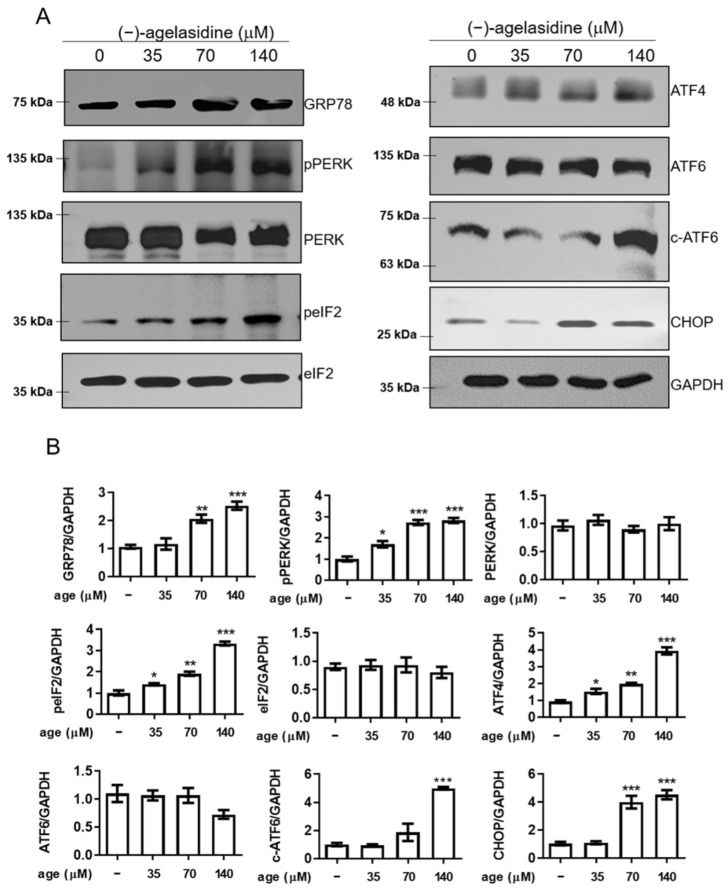
The effects of (−)-agelasidine A on the expression of ER stress signaling pathway-related proteins in Hep3B cells. Cells were treated with different concentrations of (−)-agelasidine A for 12 h. (**A**) The cell lysates were prepared, and the expression levels of ER stress-related molecules were assessed by Western blot with whole-cell lysates. GAPDH was used as an internal control. (**B**) Quantitation of Western blot signal intensities. All data presented are representative of three independent experiments with similar results. Significant differences compared to DMSO-treated control group are indicated by * *p* < 0.05, ** *p* < 0.01, *** *p* < 0.001.

**Figure 8:**
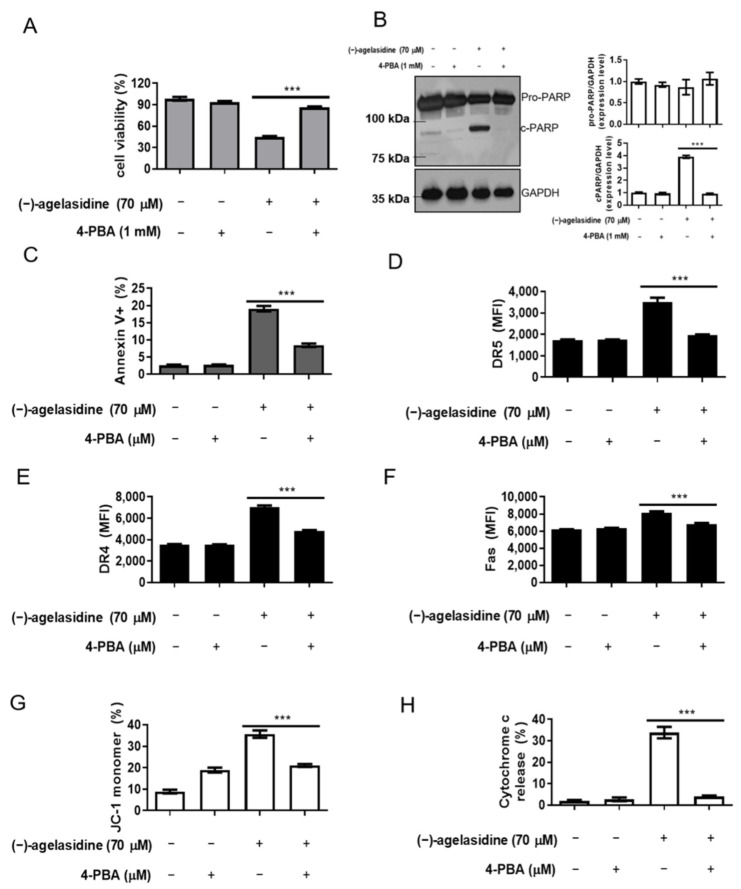
The effects of ER stress inhibitor, 4-PBA, on apoptosis and related molecules induced by (−)-agelasidine A in Hep3B cells. The cells were pretreated with 1 mM 4-PBA for 2 h, and then treated with 70 μM (−)-agelasidine A for a further 24 h. (**A**) The cell viability was measured by MTT assay. (**B**) Expression levels of cleaved poly (ADP-ribose) polymerase (PARP) were investigated by Western blotting using GAPDH as a loading control. (**C**) Phosphatidylserine externalization and DNA integrity were determined by FITC/annexin-V and PI, respectively. (**D**–**F**) DR5, DR4, and FAS levels were determined via flow cytometry. (**G**) Mitochondrial membrane potential (Δψm) and (**H**) cytochrome C release were determined by JC-1 fluorescent dye staining or anti-cytochrome c –FITC antibody and flow cytometry analysis. Data are presented as the mean ± SD in three replicates for each treatment. All data presented are representative of three independent experiments with similar results. Significant differences between (−)-agelasidine A only and 4-PBA + (−)-agelasidine A treatment are indicated by *** *p* < 0.001 (Student’s *t*-test).

Since cellular apoptosis can be driven by long periods of ER stress [16,17], we further looked into whether (−)-agelasidine A could activate this pathway. Proteins associated with ER stress were investigated, including phospho-PKR-like ER kinase (p-PERK), phospho-eukaryotic initiation factor-2α (p-eIF2α), ATF-4, cleaved ATF-6, and CHOP. Hep3B cells were treated with increasing doses of (−)-agelasidine A for 12 h. Experiments showed that (−)-agelasidine A induced the expressions of ER stress markers, p-PERK, p-eIF2α, ATF-4, cleaved ATF-6, and CHOP, in HCC in a dose-dependent manner (Figure 7A,B). Flow cytometry experiments showed that, after 2 h of pretreatment with the ER stress inhibitor, sodium 4-phenylbutyrate (4-PBA), the (−)-agelasidine A-mediated reduction in cell viability was suppressed (Figure 8A) compared to cells treated with (−)-agelasidine A only. Flow cytometry also revealed that 4-PBA-treated cells had decreased cleavage of PARP (Figure 8B), annexin V+ apoptosis (Figure 8C, Supplementary Figure S1A), and death receptor DR5, DR4, and FAS expression (Figure 8D–F, Supplementary Figure S1B), as well as mitochondrial dysfunction (Figure 8G, Supplementary Figure S1C) and cytochrome c release (Figure 8H, Supplementary Figure S1D) compared to (−)-agelasidine A only. These results show that (−)-agelasidine A efficiently activated ER stress in HCC, which in turn initiated cell apoptosis.

## 3. Discussion

HCC is one of the most frequently diagnosed malignancies, and the vast majority of patients are diagnosed too late to receive surgeries. In this way, many of the patients need alternative options such as chemotherapies and target therapies. Sorafenib and lenvatinib, tyrosine kinase inhibitors that target angiogenesis in cancer cells, are the currently licensed treatment options for metastatic HCC, HCC ineligible for surgeries, and HCC cases unable to undergo focal therapies. However, treatment effects are sometimes unreliable, and the average recovery time of treated patients is around 8 months and 4 months with lenvatinib and sorafenib treatment, respectively [18]. Exploration of anticancer drugs that are safer and more efficient is essential for the progress of modern medicine.

**Figure 9:**
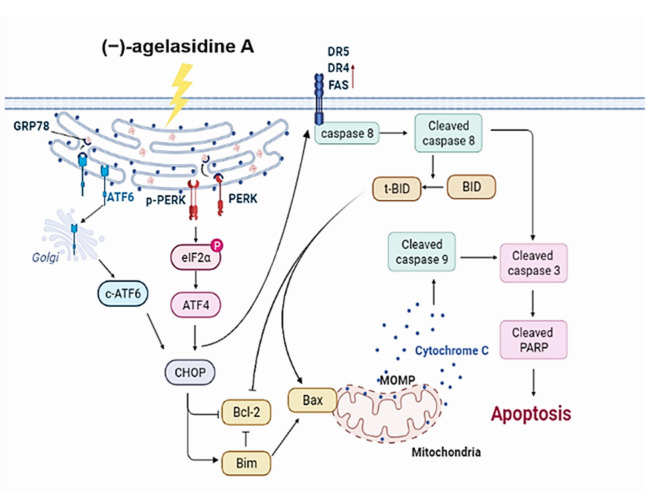
Proposed model of mechanisms exploited by (−)-agelasidine A to induce apoptosis in Hep3B liver cancer cells. A mechanism involving endoplasmic reticulum stress is outlined for the (−)-agelasidine A -mediated apoptosis via both intrinsic mitochondrial and extrinsic pathways. With this mechanism, (−)-agelasidine A leads to a damaged mitochondrial membrane in Hep3B cells, as well as upregulated protein expression of CHOP, cleaved ATF-6, death receptor (DR4/5, FAS), cleaved caspase-8, cleaved BID, Bim, Bax, cytochrome c, cleaved caspase-9, and cleaved caspase-3, thus triggering cell apoptosis.

Ocean invertebrates offer a diverse source of metabolites with anticancer activities, and many of them are licensed for cancer treatment [5,19]. (−)-Agelasidine A, an alkaloid extracted from *Agelas* *nakamurai*, has been shown to have antifungal abilities [17], yet its anticancer abilities have not been investigated. In this study, through in vitro experiments, the anticancer abilities of (−)-agelasidine A were demonstrated. On the basis of our current understanding, (−)-agelasidine A induces ER stress followed by apoptosis to produce anticancer effects. Figure 9 demonstrates the proposed mechanism of (−)-agelasidine A-driven apoptosis in Hep3B cell lines.

Apoptosis is a process where dysfunctional or damaged cells undergo programmed cell death, and it is an essential mechanism via which organisms maintain homeostasis [20]. Apoptosis is also an essential pathway responsible for eradication of cancerous cells that arise in the body. In this study, Hep3B cellular apoptosis was promoted after introducing (−)-agelasidine A. Considering that there are intrinsic and extrinsic pathways in the regulation of cellular apoptosis, we examined the proapoptotic function of (−)-agelasidine A according to signals in each pathway (Figure 9). The intrinsic pathway is initiated by the loss of MMP and mitochondrial membrane perturbation. In this process, complexes of antiapoptotic Bcl-2 and Bad stimulate the release of proapoptotic Bax, while permeability of the mitochondria is altered by Bim. This causes the release of cytochrome c from mitochondria to the cytoplasm. Subsequently, executioner caspase-3/PARP is activated by apoptotic bodies. The extrinsic pathway is driven by the formation of death-inducing signaling complex (DISC) and activation of caspase-8. After proapoptotic tBID is processed by caspase-8, it binds to antiapoptotic Bcl-2 and upregulates the release and activity of proapoptotic Bax. Consequently, the extrinsic pathway can be connected to the intrinsic pathway. In our experiment, (−)-agelasidine A could promote apoptosis via the intrinsic pathway, including inducing proapoptotic Bax and Bim, reducing antiapoptotic Bcl-2, altering MMP, facilitating release of cytochrome c, and enhancing executioner caspase-9. By increasing death receptors (DRs) such as DR4, DR5. and FAS, (−)-agelasidine A could also upregulate caspase-8 and tBID and, consequently, stimulate both extrinsic and intrinsic pathways (Figure 6). Our inhibitory experiment with caspase-3, -8, and -9 inhibitors also indicated the multiple possible mechanisms of action of (−)-agelasidine A in inducing apoptosis in cancerous cells regardless of intrinsic and extrinsic pathways.

The ER is essential in maintaining cellular homeostasis and inducing cytotoxic cellular death. Its function can be disrupted by different pathophysiological conditions that cause the accumulation of unfolded proteins. This results in ER stress and leads to activation of the unfolded protein response (UPR). The UPR can then initiate cellular apoptosis. In mammal cells, UPR caused by ER stress is regulated by three ER transmembrane proteins, which are ATF6, PERK, and IRE1α [21,22]. Emerging evidence shows that natural compounds and their derivatives have the potential to treat cancers, metabolic diseases, cardiovascular diseases, and neurodegenerative disorders by regulating ER stress-related pathways [23,24,25]. In our study, (−)-agelasidine A could induce ER stress in dose-dependent and time-dependent fashions. It also demonstrated that ER-stress-related proteins such as GRP78, p-PERK, p-eIF2α, ATF4, c-ATF6, and CHOP are all upregulated during treatment. Through inhibitor-driven CHOP suppression, our studies succeeded in reducing ER stress-induced cellular apoptosis. In our experiment, adding 4-PBA to inhibit ER stress could suppress DRs, as well as accomplish MMP loss and release of cytochrome c. Decreased cellular apoptosis was also noted. These results suggest that (−)-agelasidine A initiated cellular apoptosis through enhancing ER stress in HCC.

In the past 20 years, marine invertebrates have been unveiled as important, prolific sources of bioactive secondary metabolites. Many of these compounds have shown potential as anticancer medications worth further research [26]. However, although the efficacy of these marine natural compounds in fighting diseases has been distinguished, the sources are limited, and only finite amounts of natural products can be produced. Therefore, difficulties arise when trying to acquire a sufficient supply for drug discovery and development. Moreover, in the long run, collecting bioactive agents from marine invertebrates is not practical for clinical trials and mass production for pharmaceutical applications. Accordingly, the production of these potential natural pharmaceuticals via artificial methods is a more feasible means of developing such medications. Another potential approach to overcome the limited quantities of compounds from natural sources is through aquaculture of marine organisms by optimizing their growth environment in cultured tanks or pools [27,28]. A final potentially useful way to provide these medicinally attractive compounds is through chemical synthesis. With great advances in chemical synthesis, steady and controllable manufacturing processes could effectively increase the production of bioactive natural compounds and allow us to improve bioavailability by modifying their structures [29,30]. For the supply of (−)-agelasidine A for research and drug development, this barrier is less significant since 1227 mg of this compound could be isolated from 5 kg of the sponge *A. clathrodes* [17]. Thus, sufficient amounts of (−)-agelasidine A can be provided for medicinal application if aquaculture of this sponge can be achieved in the future. Furthermore, racemic agelasidine A was synthesized in 1992 [31] from commercially available farnesol via an efficient three-step synthesis. In 2019, optically active compound (−)-agelasidine A was successfully and efficiently synthesized by catalytic hydrothionation of β-farnesene [32]. It was also synthesized by regioselective and enantioselective synthesis based on palladium-catalyzed allylic sulfonylation from the tertiary allylic carbonate of farnesonol [33]. Thus, once (−)-agelasidine A is proven to be an effective agent for inhibition of in vivo HCC, this compound can be readily provided for further clinical trials of HCC treatment.

## 4. Material and Methods

### 4.1. Cell Culture

The HepG2 and Hep3B cancerous cell lines were purchased from the Food Industry Research and Development Institute (Hsinchu City, Taiwan). All cell lines were maintained in Dulbecco’s modified Eagle’s medium (DMEM), supplemented with 10% heat-inactivated fetal bovine serum (FBS), 100 U/mL penicillin, and 100 g/mL streptomycin. Cells were incubated at 37 °C in a humidified atmosphere containing 5%/95% of CO_2_/air. Cell culture reagents were purchased from Invitrogen Life Technologies (Carlsbad, CA, USA).

### 4.2. Chemicals

The frozen bodies of *Agelas nakamurai*, collected from the coast of Orchid Island (Taiwan) in 2008 and identified by Dr. Meng-Chen Yu at Biodiversity Research Center, Academia Sinica, (Taipei, Taiwan), were sliced and extracted exhaustively with ethyl acetate (EtOAc). The combined EtOAc extract was evaporated under reduced pressure to give a crude residue, which was subjected to silica gel column chromatography, eluting with EtOAc in *n*-hexane (0–100%, stepwise) and then with MeOH in acetone (0–100%, stepwise), to yield 19 fractions (F1–F19). Fraction 13 was chromatographed by elution with MeOH–acetone (3:1) to yield a partially pure compound (−)-agelasidine A. This compound was purified by chromatography over a reversed-phase C18 column using MeOH–H2O (4:1) as the eluent to afford pure (−)-agelasidine A, which was subsequently confirmed by NMR and specific optical rotation, both of which appeared to be identical with data reported previously ([α]${}_{D}^{25}$ −24.9 and Figure S2 [17]).

### 4.3. Cell Viability Assay

Cancerous cells, Hep3B and HepG2, were seeded into 24-well plates at 2 × 10^4^ cells/well and treated with various concentrations of (−)-agelasidine A or 0.1% DMSO as a control vehicle for 24 h. Following incubation, 10 μL of 3-(4,5-dimethylthiazol-2-yl)-2,5-diphenyltetrazolium bromide (MTT, Sigma-Aldrich, St. Louis, MO, USA) solution (0.5 mg/mL final concentration) was added to each well. The supernatant was aspirated after 4 h, and then 600 μL of DMSO was added to dissolve the formazan crystals. The absorbance was examined at 540 nm using a microplate reader (TECAN, Durham, NC, USA). Data were shown as the percentage absorbance of (−)-agelasidine A-treated cells relative to DMSO-treated cells. The 50% inhibitory concentration (IC_50_) values were calculated using GraphPad software for semi-log curve fitting with regression analysis. This cell viability assay was also performed to examine the involvement of the caspase pathway or ER stress in the (−)-agelasidine A-mediated cytotoxicity. Briefly, Hep3B cells were incubated overnight and then pretreated with 1 mM 4-PBA (Cayman Chemical Company, Ann Arbo, MI, USA), caspase-3 inhibitor (Z-DEVD-FMK, 10 µM, R&D Systems, Minneapolis, MN, USA), caspase-8 inhibitor (Z-IETD-FMK, 20 µM, BioVision, San Francisco, CA, USA), or caspase-9 inhibitor (Z-LEHD-FMK, 20 µM, BioVision, Milpitas, CA, USA) for 2 h. Cells were then treated with 70 μM (−)-agelasidine A for an additional 24 h prior to MTT assay.

Normal hepatocytes were also isolated by in situ retrograde collagenase perfusion as described previously [34] and tested for cytotoxicity of (−)-agelasidine A with MTT assay. Briefly, hepatocytes were dissociated from the collagenase-digested liver after PBS perfusion and filtered through a gauze. The cell suspension was then fractionated by Percoll density centrifugation (2500 rpm for 5 min at 4 °C) to purify hepatocytes, followed by incubating at 37 °C under an atmosphere of 95% air–5% CO_2_ and testing via MTT assay.

### 4.4. Colony-Forming Assays

Colony formation assays were carried out to test the effect of (−)-agelasidine A on the clonogenicity of HepG2 and Hep3B cells. Briefly, cells were seeded into six-well plates at 500 cells/well and incubated for 24 h. The cells were then treated with different concentrations of (−)-agelasidine A (35, 70, and 140 μM) for 1 week to allow colonies to form. Crystal violet (2%) (Sigma-Aldrich, St. Louis, MO, USA) was used to stain colonies, and the number of colonies in each well was counted under an inverted microscope (Olympus, Tokyo, Japan).

### 4.5. Analysis of Cell Apoptosis by Flow Cytometry

The apoptosis assay was performed using an annexin V/FITC apoptosis detection kit (BD Biosciences, Franklin Lakes, NJ, USA). The Hep3B cells (2 × 10^5^/well) were seeded in six-well plates and incubated overnight. After treatment with (−)-agelasidine A (0, 35, 70, or 140 µM) for 24 h, cells were harvested and washed in PBS. Cells were incubated with 5 μL of annexin V/FITC (20 μg/mL) and 5 μL of propidium iodide (PI) (50 μg/mL) at room temperature for 10 min in the dark. Apoptotic cells were detected using an AccuriTM C5 cytometer (BD Biosciences, Franklin Lakes, NJ, USA), and the data were analyzed using BD Accuri C6 Software version 1.0.264.21 (BD Biosciences, Ann Arbor, MI, USA).

### 4.6. Determination of DNA Content by Flow Cytometry

Hep3B cells were seeded into six-well plates at a density of 2 × 10^5^ cells/well and treated with (−)-agelasidine A (35, 70, and 140 μM) for 24 h. The cells were harvested and fixed with 70% ethanol overnight at −20 °C. Subsequently, cells were stained with PI/RNase staining buffer (BD Biosciences, San Diego, CA, USA) in PBS for 30 min. DNA content was analyzed using an Accuri C5 cytometry (BD Biosciences, Ann Arbor, MI, USA).

### 4.7. Western Blot Analysis 

Cells were seeded into six-well plates at a density of 2 × 10^5^ cells/well and treated with indicated concentrations of (−)-agelasidine A for 12 h (for ER stress molecules) or 24 h. The cells were collected, and cell pellets were lysed in RIPA buffer containing 1% protease inhibitor cocktail (Sigma-Aldrich, St. Louis, MO, USA) and phosphatase inhibitor (Roche, Mannheim, Germany). The BCA protein assay (Thermo Fisher Scientific, Waltham, MA, USA) was used for quantitation of total protein. An equal amount of protein was immobilized by 5–12% sodium dodecyl sulfate (SDS) polyacrylamide gel electrophoresis and transferred onto polyvinylidene difluoride membranes (Merck Millipore, Billerica, MA, USA). The membranes were then blocked in BlockPRO Protein-Free Blocking Buffer (Visual protein, Taipei City, Taiwan) for 1 h, followed by incubation with anti-PARP (Polyconal, 1:1000; Cell Signaling Technology, Danvers, MA, USA), anti-Bim (clone C34C5, 1:1000; Cell Signaling Technology,) anti-Bax (clone 2D2, 1:3000; BioLegend, San Diego, CA, USA), anti-Bcl-2 (clone 100, 1:2000; BioLegend, San Diego, USA), anti-Bid (Clone 2002, 1:1000; Cell Signaling Technology, Danvers, MA, USA), anti-GRP78 (Polyclonal, 1:10,000; GeneTex Inc., Irvine, CA, USA), anti-phospho-PERK(Thr 980) (Polyclonal, 1: 1000; Bioss, Woburn, MA, USA), anti-PERK (Clone D11A8, 1:1000; Cell Signaling Technology, Danvers, MA, USA), anti-Phospho-eIF2α (Clone 119A11, 1:1000; Cell Signaling Technology, Danvers, MA, USA), anti- eIF2 (polyconal, 1:1000; Cell Signaling Technology, Danvers, MA, USA), ATF4 (clone D4B8, 1:1000; Cell Signaling Technology, Danvers, MA, USA); ATF6 (clone W17028A, 1:1000; BioLegend, San Diego, CA, USA), anti-CHOP (clone L63F7, 1:1000; Cell Signaling Technology, Danvers, MA, USA), and glyceraldehyde 3-phosphate dehydrogenase (GAPDH) (clone 6C5, Abcam, Cambridge, MA, USA) antibodies at 4 °C overnight. The next day, the membrane was incubated with the horseradish peroxidase (HRP)-conjugated corresponding secondary antibodies (Jackson ImmunoResearch Laboratories, West Grove, PA, USA) at 4 °C overnight. After washing with TBST, the immunoreactive band was detected using LumiFlash Ultima Chemiluminescent substrate in an HRP system (Visual protein, Taipei City, Taiwan; LF08–500) and imaged using a Hansor Luminescence Image System (Taichung, Taiwan). The band densities were analyzed using the ImageJ 1.47 program from the National Institute of Health (NIH) (Bethesda, MD, USA).

### 4.8. Caspase Activity Assay by Flow Cytometry

The cells were seeded into six-well plates at a density of 2 × 10^5^ cells/well and treated with (−)-agelasidine A (35, 70, and 140 μM) for 24 h. Caspase-3, -8, and -9 activities were measured using the appropriate CaspGLOW fluorescein active caspase staining kits (Biovision, Milpitas, CA, USA) according to the manufacturer’s protocol and measured by flow cytometry on an Accuri C5 cytometry (BD Biosciences, Ann Arbor, MI, USA).

### 4.9. Measurement of Mitochondrial Membrane Potential

Mitochondrial membrane potential (MMP) was detected using JC-1 dye (Invitrogen Life Technologies, Carlsbad, CA, USA). Briefly, Hep3B cells were treated with (−)-agelasidine A (35, 70, and 140 μM) for 24 h, collected, and washed with PBS. Then, the cells were stained with JC-1 fluorescent dye at 37 °C for 20 min in the dark. After incubation, the level of mitochondrial membrane potential (ΔΨm) was determined using an Accuri C5 cytometer (BD Biosciences, Ann Arbor, MI, USA). JC-1 monomers and J-aggregates were detected in the FL1 and FL2 channels, respectively, whereby variations in the red/green fluorescence intensity ratio reflected changes in the mitochondrial membrane potential.

### 4.10. In Vitro Assay for Cytochrome c Release from Mitochondria by Flow Cytometry

The release of cytochrome c from the mitochondria to the cytosol was detected by FITC–anti-cytochrome c antibody (Clone 6H2.B4, 1:1000; BioLegend, San Diego, CA, USA). Briefly, cells were seeded into six-well plates (2 × 10^5^ cells/well) and treated with the indicated concentrations of (−)-agelasidine A for 24 h. Subsequently, cells were permeabilized with 100 µL of digitonin lysis buffer (50 µg/mL digitonin and 100 mM KCl in 1× PBS) for 5 min on ice and then fixed in 4% paraformaldehyde (Sigma-Aldrich, St. Louis, MO, USA) in PBS for 20 min at RT. Cells were stained with FITC–anti-cytochrome c antibody in 0.5 mL of labeling buffer (2% BSA in 0.05% Triton X-100 PBS) at 4 °C for 35 min, and then detected for cytochrome c-positive cells by Accuri C5 cytometry (BD Biosciences, Ann Arbor, MI, USA).

### 4.11. Statistical Analysis

All data were presented as the means ± standard deviation (SD), and two-tailed Student’s *t*-tests were used in examining the null hypothesis of two unpaired independent samples. One-way ANOVA was performed to compare different groups using GraphPad Prism Version 6.0 (San Diego, CA, USA). The acceptable type 1 error was considered to be 5%.

## 5. Conclusions

In conclusion, (−)-agelasidine A is able to reduce the viability and growth of human HCC and Hep3B cells. Moreover, intrinsic and extrinsic apoptosis can be initiated by (−)-agelasidine A-induced ER stress. In this way, (−)-agelasidine A is deemed a potential candidate for treating HCC.

## Data Availability

The data that support the findings of this study are available from the corresponding author upon reasonable request.

## Supplementary Materials

The following supporting information can be downloaded at: https://www.mdpi.com/article/10.3390/md20020109/s1, Figure S1: The effects of ER stress inhibitor, 4-PBA on the apoptosis and apoptotic related molecules induced by (−)-agelasidine A in Hep3B cells; Figure S2: The optical rotation of this isolated compound was [α]${}_{D}^{25}$ −24.9 (c 1.0, MeOH) with a molecular formula C18H33N3SO2 (ESIMS: *m*/*z* 356 [M + H]^+^).

## References

[1] Siegel R.L., Miller K.D., Jemal A. (2019). Cancer statistics, 2019. CA Cancer J. Clin..

[2] Zhu J., Yin T., Xu Y., Lu X.J. (2019). Therapeutics for advanced hepatocellular carcinoma: Recent advances, current dilemma, and future directions. J. Cell. Physiol..

[3] Longo D., Villanueva A. (2019). Hepatocellular carcinoma. N. Engl. J. Med..

[4] Llovet J.M., Kelley R.K., Villanueva A., Singal A.G., Pikarsky E., Roayaie S., Lencioni R., Koike K., Zucman-Rossi J., Finn R.S. (2021). Hepatocellular carcinoma. Nat. Rev. Dis. Primers.

[5] Khalifa S.A., Elias N., Farag M.A., Chen L., Saeed A., Hegazy M.-E.F., Moustafa M.S., El-Wahed A., Al-Mousawi S.M., Musharraf S.G. (2019). Marine natural products: A source of novel anticancer drugs. Mar. Drugs.

[6] Demain A.L., Vaishnav P. (2011). Natural products for cancer chemotherapy. Microb. Biotechnol..

[7] Newman D.J., Cragg G.M. (2020). Natural products as sources of new drugs over the nearly four decades from 01/1981 to 09/2019. J. Nat. Prod..

[8] Pandey A. (2019). Pharmacological significance of marine microbial bioactive compounds. Environ. Chem. Lett..

[9] Hu Y., Chen J., Hu G., Yu J., Zhu X., Lin Y., Chen S., Yuan J. (2015). Statistical research on the bioactivity of new marine natural products discovered during the 28 years from 1985 to 2012. Mar. Drugs.

[10] Dyshlovoy S.A., Honecker F. (2019). Marine Compounds and Cancer: The First Two Decades of XXI Century. Mar. Drugs.

[11] Bian C., Wang J., Zhou X., Wu W., Guo R. (2020). Recent advances on marine alkaloids from sponges. Chem. Biodivers..

[12] Berlinck R.G., Trindade-Silva A.E., Santos M.F. (2012). The chemistry and biology of organic guanidine derivatives. Nat. Prod. Rep..

[13] Rubiolo J., López-Alonso H., Roel M., Vieytes M., Thomas O., Ternon E., Vega F., Botana L. (2014). Mechanism of cytotoxic action of crambescidin-816 on human liver-derived tumour cells. Br. J. Pharmacol..

[14] Nakamura H., Wu H., Kobayashi J., Ohizumi Y., Hirata Y., Higashijima T., Miyazawa T. (1983). Agelasidine-A, a novel sesquiterpene possessing antispasmodic activity from the okinawa sea sponge *Agelas* sp.. Tetrahedron Lett..

[15] Nakamura H., Wu H., Kobayashi J., Kobayashi M., Ohizumi Y., Hirata Y. (1985). Agelasidines: Novel hypotaurocyamine derivatives from the Okinawan sea sponge *Agelas nakamurai* Hoshino. J. Org. Chem..

[16] Capon R.J., Faulkner D.J. (1984). Antimicrobial Metabolites from a Pacific Sponge, *Agelas* sp.. J. Am. Chem. Soc..

[17] Medeiros M.A., Lourenço A., Tavares M.R., Curto M.J.M., Feio S.S., Roseiro J.C. (2006). (−)-Agelasidine A from *Agelas clathrodes*. Zeitschrift für Naturforschung C.

[18] Huang A., Yang X.-R., Chung W.-Y., Dennison A.R., Zhou J. (2020). Targeted therapy for hepatocellular carcinoma. Signal Transduct. Target. Ther..

[19] Ercolano G., de Cicco P., Ianaro A. (2019). New drugs from the sea: Pro-apoptotic activity of sponges and algae derived compounds. Mar. Drugs.

[20] Wong R.S. (2011). Apoptosis in cancer: From pathogenesis to treatment. J. Exp. Clin. Cancer Res..

[21] Logue S.E., Cleary P., Saveljeva S., Samali A. (2013). New directions in ER stress-induced cell death. Apoptosis.

[22] Wei J., Fang D. (2021). Endoplasmic Reticulum Stress Signaling and the Pathogenesis of Hepatocarcinoma. Int. J. Mol. Sci..

[23] Limonta P., Moretti R.M., Marzagalli M., Fontana F., Raimondi M., Montagnani Marelli M. (2019). Role of Endoplasmic Reticulum Stress in the Anticancer Activity of Natural Compounds. Int. J. Mol. Sci..

[24] Choy K.W., Murugan D., Mustafa M.R. (2018). Natural products targeting ER stress pathway for the treatment of cardiovascular diseases. Pharm. Res..

[25] Pereira D.M., Valentão P., Correia-da-Silva G., Teixeira N., Andrade P.B. (2015). Translating endoplasmic reticulum biology into the clinic: A role for ER-targeted natural products?. Nat. Prod. Rep..

[26] Varijakzhan D., Loh J.-Y., Yap W.-S., Yusoff K., Seboussi R., Lim S.-H.E., Lai K.-S., Chong C.-M. (2021). Bioactive compounds from marine sponges: Fundamentals and applications. Mar. Drugs.

[27] Gomes N.G., Dasari R., Chandra S., Kiss R., Kornienko A. (2016). Marine invertebrate metabolites with anticancer activities: Solutions to the “supply problem”. Mar. Drugs.

[28] Pomponi S.A. (1999). The Bioprocess-Technological Potential of the Sea, in Progress in Industrial Microbiology.

[29] Smith III A.B., Freeze B.S. (2007). (+)-Discodermolide: Total synthesis, construction of novel analogues, and biological evaluation. Tetrahedron.

[30] Florence G.J., Gardner N.M., Paterson I. (2008). Development of practical syntheses of the marine anticancer agents discodermolide and dictyostatin. Nat. Prod. Rep..

[31] Ichikawa Y., Kashiwagi T., Urano N. (1992). Biomimetic synthesis of agelasidine A. J. Chem. Soc. Perkin Trans..

[32] Yang X.H., Davison R.T., Nie S.Z., Cruz F.A., McGinnis T.M., Dong V.M. (2019). Catalytic Hydrothiolation: Counterion-Controlled Regioselectivity. J. Am. Chem. Soc..

[33] Um H.S., Min J., An T., Choi J., Lee C. (2018). Stereoselective allylic reduction via one-pot palladium-catalyzed allylic sulfonation and sulfinyl retro-ene reactions. Org. Chem. Front..

[34] Mederacke I., Dapito D.H., Affò S., Uchinami H., Schwabe R.F. (2015). High-yield and high-purity isolation of hepatic stellate cells from normal and fibrotic mouse livers. Nat. Protoc..

