# Supplementary material for: (−)-Agelasidine A Induces Endoplasmic Reticulum Stress-Dependent Apoptosis in Human Hepatocellular Carcinoma
Source: Mar Drugs. 2022 Jan 29;20(2):109. doi: 10.3390/md20020109 (PMC8875608; doi:10.3390/md20020109)
Supplement: Supplementary file 1 [file marinedrugs-20-00109-s001.zip › marinedrugs-1524221-supplementary.pdf]

Figure S1.

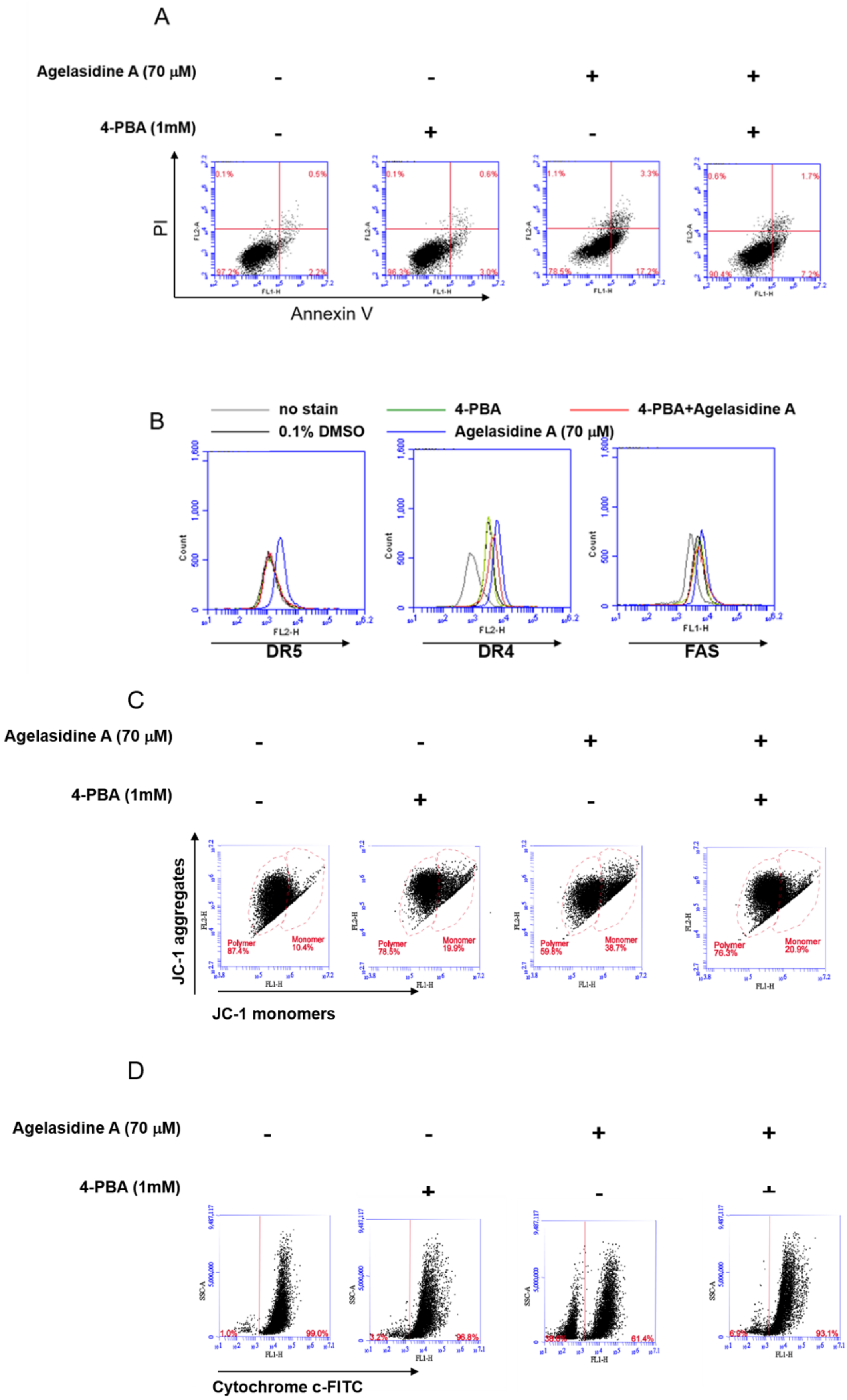
A
Agelasidine A (70 µM)
4-PBA (1mM)
PI
Annexin V
B
no stain
0.1% DMSO
4-PBA
Agelasidine A (70 µM)
4-PBA+Agelasidine A
DR5
DR4
FAS
C
Agelasidine A (70 µM)
4-PBA (1mM)
JC-1 aggregates
JC-1 monomers
D
Agelasidine A (70 µM)
4-PBA (1mM)
Cytochrome c-FITC

Figure S1. The effects of ER stress inhibitor, 4-PBA on the apoptosis and apoptotic related molecules induced by (−)-agelasidine A in Hep3B cells. Representative bivariate graphs of A) Phosphatidylserine externalization and DNA integrity were determined by FITC-annexin-V and PI. B) DR5, DR4 and FAS were determined via flow cytometry. C) Mitochondrial membrane potential (Δψm) and D) cytochrome C release was determined by JC-1 fluorescent dye staining or anti-cytochrome c –FITC antibody and flow cytometry analysis.

Figure S2.

^{13}C-NMR spectrum of (–)-Agelasidine A (methanol-*d*4)

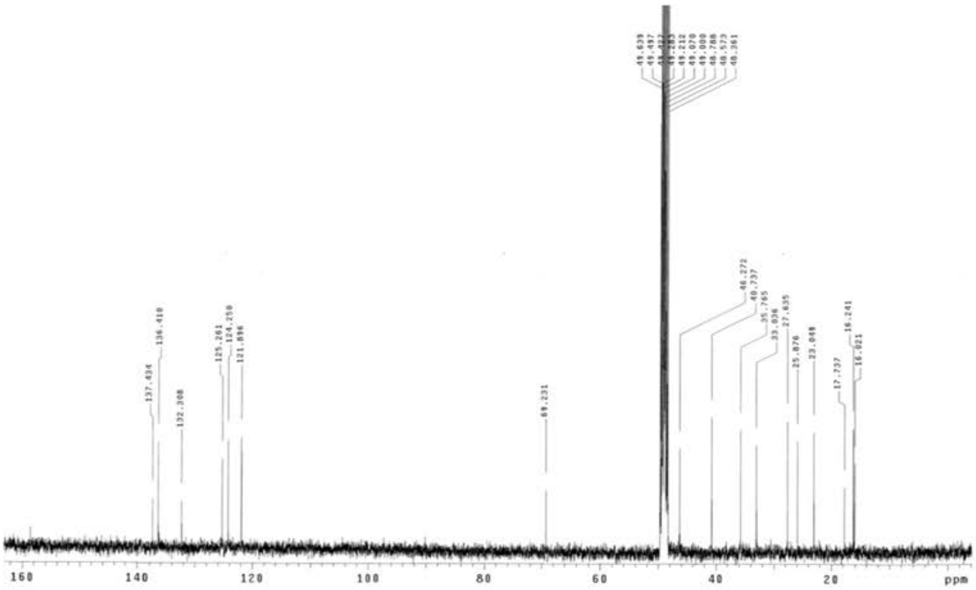

^{1}H-NMR spectrum of (–)-Agelasidine A (methanol-*d*4)

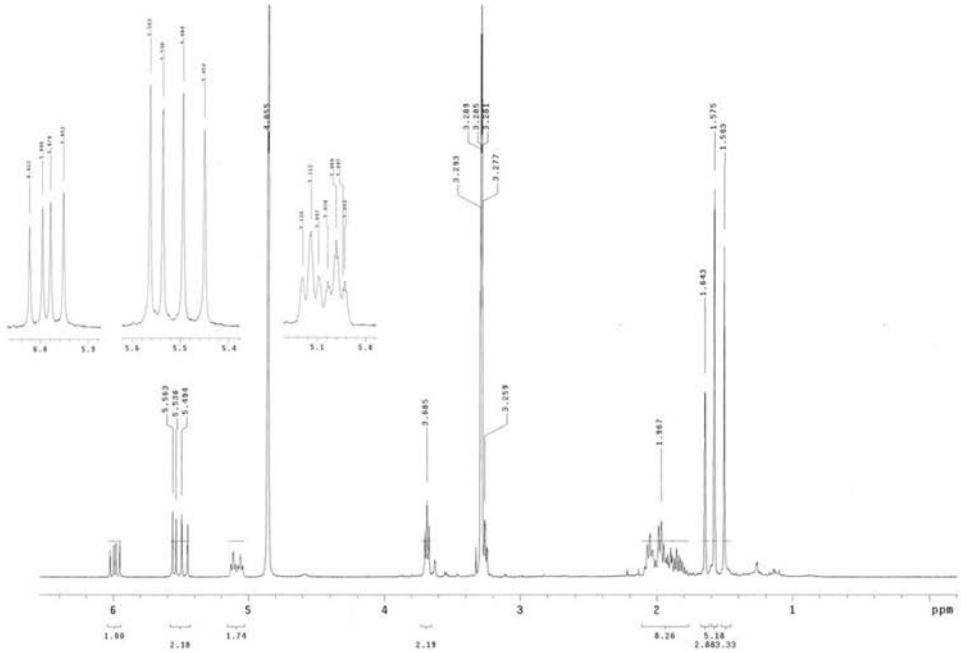

Figure S2. The optical rotation of this isolated compound was [α]25D −24.9 (c 1.0, MeOH) with a molecular formula C18H33N3SO2 (ESIMS: m/z 356 [M+H] +). The 13C NMR signals resonating at δC 158.6 (C), 137.4 (C), 136.4(CH), 132.3 (C), 125.3 (CH), 124.3(CH), 121.9 (CH2), 69.2 (C), 46.3 (CH2), 40.7 (CH2), 35.8 (CH2), 33.0 (CH2), 27.6

(CH2), 25.9 (CH3), 23.0 (CH2), 17.7 (CH3), 16.2 (CH3), and 16.0 (CH3) as well as the 1H NMR signals resonating at δH 5.99 (dd, J = 17.6, 10.8 Hz), 5.55 (dd, J = 10.8 Hz), 5.47 (d, J = 17.6 Hz), 5.11 (br t, J = 6.4 Hz), 5.06 (tt, J = 6.8, 1.6 Hz), 3.69 (t, J = 5.6 Hz), 3.25 (t, J = 5.6 Hz), 2.05 (m), 2.04 (m), 2.00 (m), 1.99 (m), 1.97 (m), 1.96 (m), 1.94 (m), 1.85 (m), 1.64 (s), 1.58 (s), 1.58 (s), 1.50 (s) were observed. Furthermore, this compound was found to be identical with the known metabloite (–)-agelasidine A, on the basis of comparison of their optical rotation [the reference: [α]20D –14.5 (c 1.55, MeOH)] and spectroscopic data.
